# Revealing oxidative stress-related genes in osteoporosis and advanced structural biological study for novel natural material discovery regarding *MAPKAPK2*

**DOI:** 10.3389/fendo.2022.1052721

**Published:** 2022-11-21

**Authors:** Yingjing Zhao, Weihang Li, Kuo Zhang, Meng Xu, Yujia Zou, Xiaotong Qiu, Tianxing Lu, Bo Gao

**Affiliations:** ^1^ Department of Critical Care Medicine, Nanjing First Hospital, Nanjing Medical University, Nanjing, Jiangsu, China; ^2^ Department of Orthopedic Surgery, Xijing Hospital, Air Force Medical University, Xi’an, China; ^3^ State Key Laboratory of Cancer Biology, Biotechnology Center, School of Pharmacy, Fourth Military Medical University, Xi’an, China; ^4^ Department of Aerospace Medical Training, School of Aerospace Medicine, Air Force Medical University, Xi’an, China; ^5^ Key Lab of Aerospace Medicine, Chinese Ministry of Education, Xi’an, China; ^6^ College of Clinical Medicine, China-Japan Union Hospital of Jilin University, Changchun, Jilin, China; ^7^ Department of Hepatic Surgery and Liver Transplantation Center, The Third Affiliated Hospital of Sun Yat-sen University, Guangzhou, China; ^8^ Guangdong Key Laboratory of Liver Disease Research, Guangdong Engineering Laboratory for Transplantation, Guangzhou, China; ^9^ Zonglian College, Xi’an Jiaotong University, Xi’an, Shaanxi, China

**Keywords:** high-throughput virtual screening, osteoporosis, WGCNA, *MAPKAPK2*, targeted therapy

## Abstract

**Objectives:**

This study aimed to find novel oxidative stress (OS)-related biomarkers of osteoporosis (OP), together with targeting the macromolecule Mitogen-activated protein kinase-activated protein kinase 2 (*MAPKAPK2*) protein to further discover potential novel materials based on an advanced structural biology approach.

**Methods:**

Gene expression profiles of GSE35958 were obtained from the Gene Expression Omnibus (GEO) database, which were included for weighted gene co-expression network analysis (WGCNA) and differential analysis to identify the most correlated module, to identify OS-related hub genes in the progression of OP. Functional annotations were also analyzed on the interested module to get a comprehensive understanding of these genes. Then, a series of advanced structural biology methods, including high-throughput screening, pharmacological characteristic prediction, precise molecular docking, molecular dynamics simulation, etc., was implemented to discover novel natural inhibitor materials against the *MAPKAPK2* protein.

**Results:**

The brown module containing 720 genes was identified as the interested module, and a group set of genes was determined as the hub OS-related genes, including *PPP1R15A*, *CYB5R3*, *BCL2L1*, *ABCD1*, *MAPKAPK2*, *HSP90AB1*, *CSF1*, *RELA*, *P4HB*, *AKT1*, *HSP90B1*, and *CTNNB1*. Functional analysis demonstrated that these genes were primarily enriched in response to chemical stress and several OS-related functions. Then, Novel Materials Discovery demonstrated that two compounds, ZINC000014951634 and ZINC000040976869, were found binding to *MAPKAPK2* with a favorable interaction energy together with a high binding affinity, relatively low hepatoxicity and carcinogenicity, high aqueous solubility and intestinal absorption levels, etc., indicating that the two compounds were ideal potential inhibitor materials targeting *MAPKAPK2*.

**Conclusion:**

This study found a group set of OS-related biomarkers of OP, providing further insights for OS functions in the development of OP. This study then focused on one of the macromolecules, *MAPKAPK2*, to further discover potential novel materials, which was of great significance in guiding the screening of *MAPKAPK2* potential materials.

## Introduction

Osteoporosis (OP) is a systemic skeletal destruction characterized by a low bone mass, degeneration of the microstructure of the bone tissue, and a high risk of bone fracture, which is one of the most severe complications of OP (1, 2). It is estimated that OP patients with hip fractures would increase from 1.7 million in 1990 to 6.3 million by 2050, which could result in continuous mental and physical pain, limitations of physical activity, low life quality, and a series of physiological side effects (3, 4). Due to the aging of the world population, the social and economic burden of OP is increasing gradually, which has become a public health crisis influencing the whole world (5). OP not only exists in elderly patients but also appears gradually in children and young adults in recent decades, indicating the trends of youth in OP. Thus, early diagnosis and timely intervention are great options to prevent the initiation and development of OP (6–8). Although studies found several potential biomarkers, each of them has displayed its own strengths and limitations (9); therefore, discovering more promising and specific biomarkers still remains an emergent task.

Oxidative stress (OS) is the imbalanced state between oxidation and antioxidant effects in the body, which mostly referred to the overexpression of reactive oxygen species (ROS) and abnormal elimination of protective products (10). Since the initial presentation of the mitochondrial theory of aging, OS has been implicated as a causative factor in many diseases including neoplasms, chronic diseases, cardiovascular diseases, neurodegenerative diseases, and chronic kidney disease. Different types of OS biomarkers have been identified, which may provide pivotal information and guide for the roles of OS in these diseases (11–14). Recent studies have pointed out that OS may also be one of the possible culprits causing functional uncoupling of osteoblasts and osteoclasts in OP (15–17). Consequently, understanding the effects of OS in the development of OP could help further enhance the preventive and therapeutic measures based on OS.

Serving as the main sources of new drugs and therapeutic agents, natural products and their structural analogs have made great contributions to the current pharmacological market, which brings advantages and challenges to the drug discovery process. Natural products also have some special features compared to the conventional synthetic molecules, such as their convertibility, malleability, and availability. Moreover, their usage in traditional medicine has provided insights focusing on safety and efficacy (18–21). Compared to typical synthetic small-molecule libraries, natural products are rich in biological activity, covering a wider range of chemical modification spaces (20, 22). Currently, advanced approaches for natural product discovery mainly include high-throughput screening technology, modern artificial intelligence, and activity-based profiling, aiming to identify more effective drugs. Among them, high-throughput screening is considered the best strategy for discovering such active substances (21). Several existing natural active inhibitors have been identified regarding specific macromolecules based on this advanced technology, such as Matrix Metallopeptidase 9 (*MMP9*) and Enhancer of Zeste Homolog 2 (*EZH2*), which all present good efficacy on different disease cell models (23, 24).

Weighted gene co-expression network analysis (WGCNA) is a holistic and systematic method for interested module integration and identification. This study combined the WGCNA method with advanced high-throughput screening technology to decipher the driver OS-related genes in the development of OP and discover potential targeted lead compounds regarding *MAPKAPK2* (also known as *MK2*). After performing comprehensive analytical methods, several differentially expressed oxidative stress genes (DEOSGs) were identified as hub OS-related genes responsible for the progression of OP, which could offer more understanding about the relationships between the OS-related genes and the development of OP; then, a series of structural biological approaches were conducted to further screen ideal lead compounds targeting specific macromolecules, including high-throughput virtual screening, molecular docking analysis, ADMET (absorption, distribution, metabolism, excretion, and toxicity) property prediction, and molecular dynamics simulation, to fully evaluate the pharmacological properties of possible natural compounds, thereby providing more choices and reserves on the targeted treatment of OP.

## Materials and methods

### Acquisition of the microarray data

Microarray data of OP patients were obtained from the Gene Expression Omnibus (GEO) database (https://www.ncbi.nlm.nih.gov/geo/), which is a powerful analytical repository including high-throughput sequencing data and microarray data. GSE35958 was designed to sequence the RNA expression profiling in human mesenchymal stem cells between normal patients and primary OP patients, including four elderly patients and five primary OP patients (25), which was well designed and was extracted from the GEO database for subsequent WGCNA and differentially expressed gene (DEG) analysis in this study.

### Preprocessing of gene expression profiling

This study downloaded raw data (‘.CEL’ file format) from GSE35958, the gene expression matrix was generated by R, followed by robust multiarray (RMA) background correction and normalization (“rma” function, “affyPLM,” “affy” packages in R), then quality control was conducted based on normalized unscaled standard error (NUSE). After the gene expression matrix was generated, the matrix was further applied to convert probe sets into gene symbols according to manufacturer-provided annotation files (GPL570), probe sets without corresponding gene symbols were removed, and the median expression values were calculated for multiple probe sets targeting one gene symbol. The preprocessed matrix file was then conducted into the following WGCNA and DEG analysis.

### Weighted gene co-expression network analysis

WGCNA was performed based on R environment (version 4.1.3) based on the “WGCNA” package (26). The top 5,000 most variable genes were extracted to construct the co-expression network (according to variance). Hierarchical clustering analysis was conducted for sample quality control; unqualified samples were eliminated from the analysis. Then, the soft threshold power was calculated to choose the most optimal value for subsequent network establishment to approximate the scale-free topology network, namely, the real biological network state. The most optimal value was chosen when the scale-free index (R^2^) reached 0.85 and the mean connectivity approached 0.

Then, the weighted gene co-expression network was established based on the most optimal soft threshold power. Different co-expression modules were identified and clustered with each other according to the similarity of each gene through “plotDendroAndColors” function. The minimum number of genes in each module was set at 30, and the maximum block size was set at 5,000. Eigengene adjacency was calculated to evaluate the interactions of different clustered modules, and the degrees of correlations between each gene and module were calculated through the topological overlap measure (TOM) to assess the most correlated module based on “selectTOM” and “TOMplot” functions (27, 28).

Module–trait correlations were also calculated to recognize the relationships between modules and clinical traits based on Pearson’s correlations. The most interested module was identified according to the lowest P value and the highest correlations. Gene significance (GS) vs. the module membership (MM) scatter plot between the interested module and clinical trait (CON and OP groups) was then calculated and visualized to validate the significance of this module, which was then chosen as the interested module and used for the following research.

### Analysis of the differentially expressed genes

The DEGs between normal and primary OP patients were analyzed and identified according to the gene expression matrix from GSE35958 using R (“limma” package). The false discovery rate and adjusted P values were performed to offer a balance between the discovery of statistically significant genes and limitations of false positives. DEGs were filtered by adjusted P value with a cutoff of <0.05 and |logFC| (fold change) >1. Volcano plot was then conducted to visualize the upregulated and downregulated DEGs between the two groups.

### Functional and pathway enrichment analysis of differentially expressed genes

The screened DEGs were applied into DAVID (Database for Annotation, Visualization and Integrated Discovery) database (https://david.ncifcrf.gov/) to get a comprehensive understanding of functional annotations and interpretations, including Gene Ontology (GO) and Kyoto Encyclopedia of Genes and Genomes (KEGG) results, which was achieved by R (“clusterProfiler” package). P < 0.05 was set as the cutoff value. Gene set enrichment analysis (GSEA; http://software.broadinstitute.org/gsea/index.jsp) was further conducted to obtain more essential biological functions or pathways that may be ignored by GO and KEGG analysis. The annotation gene sets were determined as reference information. P < 0.05, gene size >20, and |enrichment scores| (ES) >0.4 were considered statistically significant.

### Screening of hub genes among oxidative stress-related genes

To obtain OS-related genes, we retrieved “oxidative stress” keywords in the GeneCards database (https://www.genecards.org) and determined OS-related genes with relevance scores ≥7. A total of 824 genes were extracted as OS-related genes and included in this study (**Supplementary Table S1**). After identification of DEGs and the key interested module by WGCNA, the DEOSGs of OP patients were determined by taking the intersection of the three parts of the genes to generate a more reliable result based on Venn plot analysis in R (“VennDiagram” package).

### Advanced structural biology approach and small-molecule repository preparation

Discovery Studio (DS; version 4.5, BIOVIA, San Diego, CA, USA) was employed in this study for structural biology research targeting macromolecules, which is a suite of software for protein structural/functional research and drug discovery by performing structural chemical and structural biological computations based on molecular modeling and simulation environment (18). DS contains a series of advanced structural biological tools, including LibDock, CDOCKER, ADMET prediction, and molecular dynamics simulation, which may help researchers effectively find more potential lead compounds through high-throughput screening and thereby reducing the time and manual power costed by traditional drug filtering methods. This study chose ZIN15 repository (http://zinc15.docking.org/) to prepare candidate drug libraries for virtual screening, which is a free database for commercially available compounds, provided and preserved by Irwin and Sterling Laboratories in the Department of Pharmaceutical Chemistry at the University of California, San Francisco (UCSF). The ZINC15 database in total contained nearly 230 million compounds with three-dimensional (3D) structure files (29). This study finally extracted 3D molecular structures from 18,225 compounds with corresponding names and biogenic and purchasable properties and employed them in the following inhibitor discovery research.

### High-throughput virtual screening based on macromolecules

The LibDock module in DS was employed to perform high-throughput virtual screening in this study, which was widely used in the drug screening process (30). LibDock is a rigid-based docking program, which conducts polar and nonpolar probes and grids placed in the binding site to calculate the hot spots of proteins. The resolution of 2.55 Å crystal structure of *MAPKAPK2* protein was obtained and downloaded from the Protein Data Bank (PDB) database (https://www.rcsb.org/, PDB ID: 3KGA), which was docked with the potent 3-aminopyrazole ATP site inhibitor (the reference drug in this study). The macromolecule was prepared by removing crystal water and surrounding heteroatoms, followed by adding hydrogen, ionization, and energy minimization. After the ligand library was prepared from the ZINC15 database, both the *MAPKAPK2*–inhibitor complex and those 18,225 ligands were imported into the working environment of the LibDock module. The prepared docking site of *MAPKAPK2* protein was determined as the 5 Å radius around the docking site of the original inhibitor. After running the high-throughput docking program, all docked ligands were ranked and listed based on LibDock scores.

### Prediction of pharmacological characteristics

To further predict the pharmacological properties of the candidate compounds from the LibDock module, this study performed ADMET prediction analysis to evaluate the characteristics of each ligand, including the aqueous solubility level, blood–brain barrier (BBB) penetration level, cytochrome P450 2D6 (CYP2D6) binding level, hepatotoxicity level, human intestinal absorption level, and plasma protein binding (PPB) level. The TOPKAT module was then applied to predict the safety of ligands, such as rodent carcinogenicity, Ames mutagenicity, weight of evidence carcinogenicity, and developmental toxicity potential. These characteristics were fully considered when screening the potential lead compounds targeting *MAPKAPK2*.

### Analysis of the molecular docking and chemical bond interactions

To perform a more precise docking algorithm, this study then conducted CDOCKER module between the candidate compounds and macromolecule protein, which is a high-precision method based on Chemistry at Harvard Macromolecular Mechanics (CHARMM) force field for ligands and receptors to generate more accurate conformations. During the docking procedure, the structures of ligands could be flexibly bent, while the structure of the receptor remained rigid. The CDOCKER interaction energy of each ligand–*MAPKAPK2* complex was calculated, which reflected the binding affinity between the ligand and *MAPKAPK2*. The existing crystal water molecules were eliminated during rigid and semiflexible docking processes to ensure that the fixed water molecules would not influence the formation of the ligand–receptor complex, then hydrogen atoms were added to the protein to prove the accurate docking results of this experiment. Then, the origin inhibitor (the reference drug in this study) docked at *MAPKAPK2* was extracted and redocked into the binding site sphere to validate the dependability of the docking algorithm, which was determined by the root mean square deviation (RMSD). The 5 Å radius around the docking site of the original inhibitor with 13 Å radius sphere was determined and prepared as the docking site of *MAPKAPK2* protein. Then, the candidate compounds were docked into the prepared binding pocket of *MAPKAPK2* protein, multiple conformations were generated during the docking process, and the best posture was chosen based on the high docking scores and reasonable docking orientations.

### Molecular dynamics simulation

This study selected the best binding postures of the ligand–*MAPKAPK2* complexes among the poses calculated by the molecule docking program, which were then prepared for molecular dynamics simulation. The docked complex was put into an orthorhombic box and solvated with an explicit periodic boundary solvation water model. Sodium chloride was added to the system at an ionic strength of 0.145 to simulate the physiological environment in the body. Then, the system was subjected to the CHARMM force field and relaxed by energy minimization (500 steps of steepest descent and 500 steps of conjugated gradient). The system was slowly driven from an initial temperature of 296 K to the target temperature of 302 K for 2 ps, and equilibration simulations were run for 5 ps. Molecular dynamics production module was run for 0.5 ns (500 ps) with a time step of 1 fs. The whole simulation was performed under normal atmospheric pressure, with a constant temperature of approximately 300 K during the simulation process. The “particle mesh Ewald” (PME) algorithm was applied to calculate long-range electrostatics, and the linear constraint solver algorithm was adapted to fix all hydrogen-involved bonds. With the initial conformation setting as the reference, different conformations generated during the simulation process were considered as variable complexes. The stability of these ligand complexes were calculated and evaluated according to RMSD, energy values, and structural characteristics through the analysis trajectory protocol.

## Results

### Establishment of the weighted gene co-expression network

**Figure 1:**
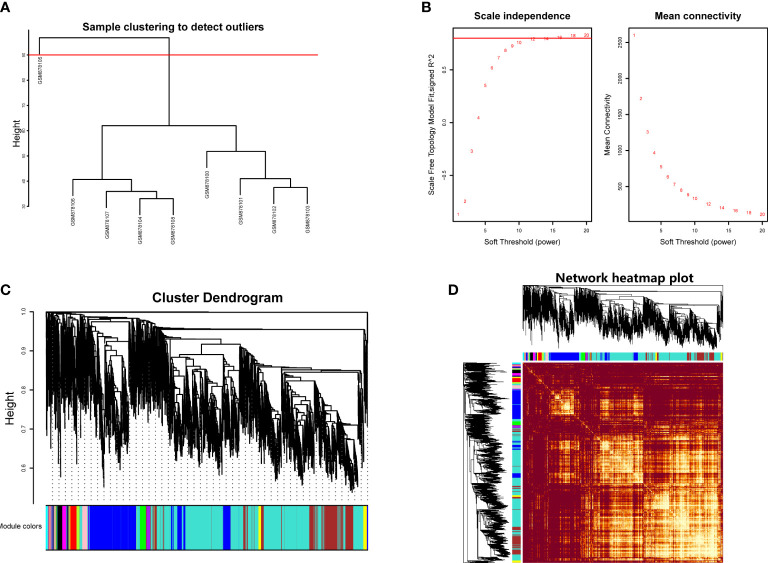
**(A)** Hierarchical clustering analysis to detect outliers in GSE35958. **(B)** Determination of soft threshold power value. Left panel represented scale-free model fit index of different values; right panel showed the mean connectivity of these values. **(C)** Dendrogram branch plot of genes based on dissimilarity measure and assignment modules. **(D)** Topological overlap measure heatmap of the weighted co-expression network. Light area indicated highly clustered modules.

Hierarchical clustering analysis was conducted to detect the heterogeneity of each sample. As shown in **Figure 1A**, GSM878105 had high heterogeneity against other samples, which were considered as the outliers and were removed. Finally, the gene expression matrix totally containing the most variable 5,000 genes and eight samples was employed for WGCNA. The soft threshold power value (β) was chosen as 16 when R^2^ reached 0.85 and the mean connectivity infinitely approached 0 (**Figure 1B**). Therefore, β was determined as 16 to establish the weighted gene co-expression network.

We then performed hierarchical clustering tree analysis based on the weighted network and gene mutual co-expression situation to cluster different genes with the similar expression patterns together. A total of 16 distinct modules were generated with the unique color based on their expression profile. The dendrogram branch plot illustrated that the genes in each module were highly heterogeneous (**Figure 1C**). Topological Overlapmatrix (TOM) analysis further validated that each identified co-expression module behaved as an independent expression pattern, which could be distinguished in the network, as shown in **Figure 1D**. Subsequently, the characteristics between clinical features and these identified modules were further analyzed in the following research.

### Interested module identification of osteoporosis

**Figure 2:**
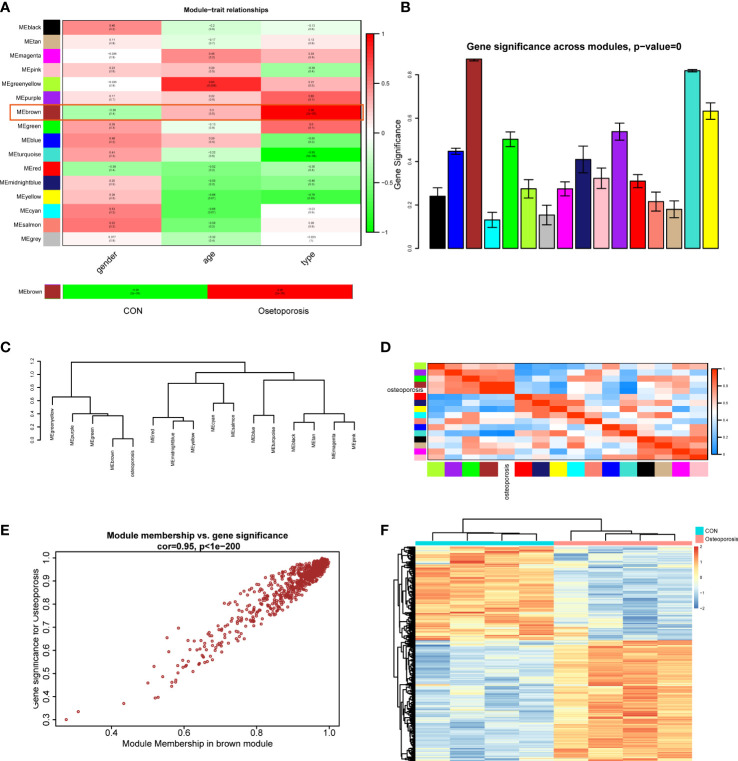
**(A)** Module–trait correlation heatmap between different clinical traits and modules. **(B)** Gene significance histogram of all clustered modules. **(C)** Cluster dendrogram of adjacencies in the eigengene network. **(D)** Heatmap of each eigenvalue in the eigengene network; blue indicated a negative correlation and red indicated a positive correlation. **(E)** Correlation scatter plot between module membership genes and gene significance in the brown module. **(F)** Expression heatmap of different groups in the brown module.

After establishment of the WGCNA network, this study then assessed the relationships between clinical traits (including gender, age, and disease type) and different modules. Correlation heatmap illustrated that all gene modules were independent from gender (all P > 0.05), genes in the greenyellow module were highly correlated with age (Cor = 0.84, P = 0.009), and genes in the brown module were positively related to disease type (Cor = 0.98, P = 2e-05), as shown in **Figure 2A**. We next extracted the brown module to analyze it with the disease type (normal and OP groups). Results indicated that in the OP group, genes in the brown module had the highest correlation (0.98) and the lowest P value (2e-05) with OP patients, elucidating that the brown module had the most correlation with OP. Therefore, the brown module was considered as the interested module, where genes could actively regulate the progression of OP. The GS histogram plot validated our conclusion and proved the reliability of the brown module (**Figure 2B**). Module eigengene adjacency was then calculated to cluster the modules and clinical traits together; the hierarchical clustering dendrogram and heatmap were used to depict the interactive roles of different eigenvalues (**Figures 2C, D**). Results suggested that the brown module and OP phenotype interacted with each other, indicating that these two eigenvalues behaved tightly and that the brown module could result in OP whether by activation or suppression of these genes.

Focusing on the brown module, we extracted the genes and performed correlations between MM (brown module) and GS; the MM-GS scatter plot illustrated that genes had a significant positive trend in the brown module (**Figure 2E**). Then, we visualized the expression patterns of those genes in the heatmap; hierarchical clustering analysis demonstrated that genes in the brown module could differentiate normal and OP groups significantly (**Figure 2F**). These results finally prompted us to choose the brown module as the key module to employ further analysis.

### Functional/pathway enrichment analysis in the brown module and confirmation of differentially expressed oxidative stress genes

**Figure 3:**
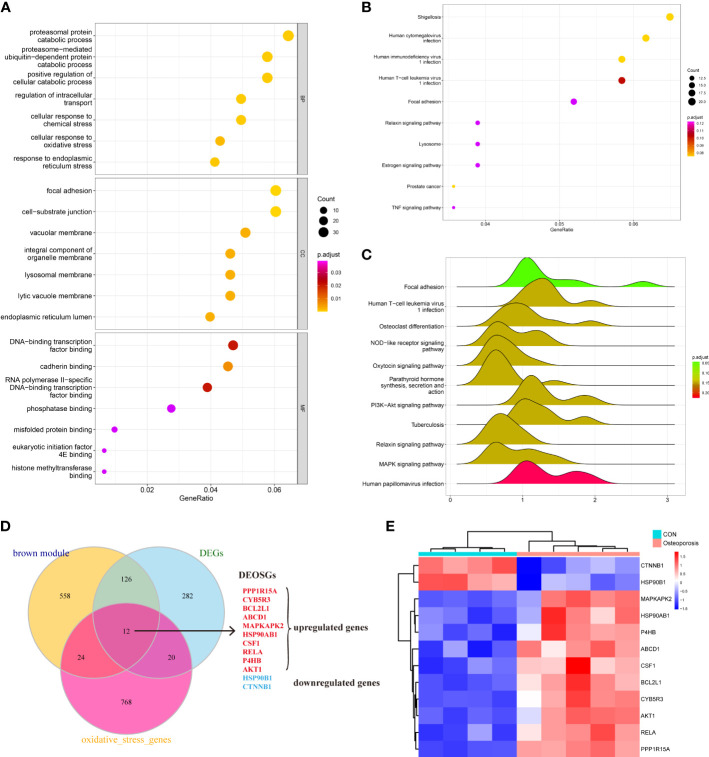
**(A–C)** Understanding the functional and pathway enrichment analysis of genes in the brown module. **(D)** Venn plot suggested that 12 genes were determined as the hub differentially expressed oxidative stress genes (DEOSGs) of osteoporosis (OP). **(E)** Heatmap of the expression patterns of these DEOSGs.

The brown module in total contained 720 genes. This study then employed GO, KEGG, and GSEA to gain further insight and information about those genes in the progression of OP. As shown in **Figure 3A**, results indicated that those active genes were highly involved in the proteasomal protein catabolic process, positive regulation of cellular catabolic process, DNA-binding transcription factor, and some OS or endoplasmic reticulum (ER) stress reactions such as the cellular response to OS, misfolded protein binding, and response to ER stress. The KEGG pathway analyzed several abnormal signaling pathways to induce OP, such as the relaxin signaling pathway and TNF signaling pathway (**Figure 3B**). Moreover, GSEA further indicated a series of valuable information about the development of OP including osteoclast differentiation, nod-like receptor signaling pathway, and *PI3K-AKT* and *MAPK* signaling pathways (**Figure 3C**). The functions and pathways mediated by these genes exhibited valuable roles, and both regulated the progression of bone homeostasis and OP, which were worth further analyzing to find novel biomarkers of OP.

After understanding the detailed functions of the brown module, we then analyzed the DEGs between normal and OP patients based on linear model fitting and Bayes algorithm, with the cutoff criteria set as P < 0.05 and |logFC| (fold change) >1. A total of 440 DEGs were identified, with 212 upregulated genes and 228 downregulated genes. Combined with the key brown module in WGCNA, DEGs, and the obtained OS-related genes, the DEOSGs were determined as the intersection of these three parts. Ultimately, 12 genes were generated, including *PPP1R15A*, *CYB5R3*, *BCL2L1*, *ABCD1*, *MAPKAPK2*, *HSP90AB1*, *CSF1*, rela, *P4HB*, *AKT1*, *HSP90B1*, and *CTNNB1*. Among them, 10 genes were upregulated and two genes were downregulated, as shown in **Figures 3D, E**. Thus, these 12 genes were considered as the DEOSGs most involved in the progression of OP, which provided a guarantee and evidence for the subsequent targeted therapy based on macromolecules.

Based on the 12 hub DEOSGs, by consulting relative literature, we found that some studies reported their inhibitor research regarding *MAPKAPK2*, indicating that the biological functions of inhibiting *MAPKAPK2* have been confirmed; however, the current exploration of drugs targeting *MAPKAPK2* is still insufficient, which blocked the discovery and reservation of compounds regarding *MAPKAPK2* in the pharmacological market. Therefore, this study further chose the *MAPKAPK2* macromolecule for further discovery of targeted inhibitors to provide more potential candidate compound reserves for medicine library.

### High-throughput virtual screening based on *MAPKAPK2*

**Figure 4:**
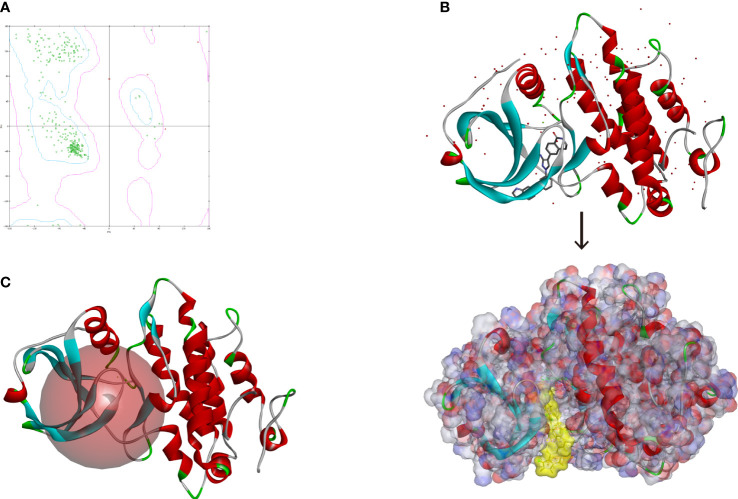
**(A)** Ramachandran diagram information of the MAPKAPK2 protein. **(B)** Docking pattern of the reference drug and MAPKAPK2 receptor. **(C)** Prepared docking site sphere of MAPKAPK2 for ligands to dock.

Altogether, 18,225 biogenic-named-purchasable natural products were obtained from the ZINC15 repository and were prepared for ligand docking. The ligand binding pocket region of *MAPKAPK2* was the pivotal regulatory site and was determined as the docking sphere for the prepared ligands to dock. Small molecules binding to the regulatory region could inhibit the active sites and thereby suppress the functions of the *MAPKAPK2* protein. The natural inhibitors targeting *MAPKAPK2* could be identified based on this mechanism. **Figure 4A** displayed the crystal structures and Ramachandran information of *MAPKAPK2* (PDB ID: 3KGA), and the docking pattern of *MAPKAPK2*-inhibitor was shown in **Figures 4B, C**. The selective 3-aminopyrazole ATP site inhibitor was chosen as the reference compound in this study, and the following docking algorithm was based on this pattern (**Figure 4C**). After high-throughput screening, 10,075 compounds could bind with residues of *MAPKAPK2* within the binding sphere. The docking scores ranged from 199.071 to 17.834, among which 276 compounds had higher LibDock scores than the reference compound (136.743). The top 30 ranked compounds were listed in **Table 1**, and all of the ligands were listed in **Supplementary Table S2** based on docking scores.

**Table 1:** Top 30 ranked materials with the highest docking scores.

Number	ZINC ID	Compounds	LibDock score
1	ZINC000062238222	5-Methyltetrahydropteroyltri-L-Glutamate	199.071
2	ZINC000085544839	Thf-Polyglutamate	186.681
3	ZINC000004096878	Coproporphyrin I	179.817
4	ZINC000085545908	Nle-Angiv	178.177
5	ZINC000095620524	N1,n5,n10,n14-Tetra-Trans-P-Coumaroylspermine	177.817
6	ZINC000004096653	Dhhpba	177.686
7	ZINC000085541163	(+-)-Grossamide	176.428
8	ZINC000004096684	Formula: C38H56O4	174.972
9	ZINC000072131515	Menaquinone	173.649
10	ZINC000004099069	S-(pga1)-Glutathione	172.841
11	ZINC000014712793	Kukoamine B	171.674
12	ZINC000150338786	Coproporphyrin Iii	169.267
13	ZINC000085826837	(+-)-Grossamide	168.849
14	ZINC000004099068	S-(pga1)-Glutathione	167.726
15	ZINC000014951658	Endomorphin 1	167.058
16	ZINC000044086691	Deoxycalyxin A	166.834
17	ZINC000049878197	N-Tricosanoyltryptamine	166.611
18	ZINC000085826835	(+-)-Grossamide	165.901
19	ZINC000008552163	E432	165.583
20	ZINC000008552069	Thf-L-Glutamate	165.453
21	ZINC000042805482	Grossamide	164.83
22	ZINC000013513540	1,14-Bis(dihydrocaffeoyl)spermine	164.111
23	ZINC000040976869	Tetrahydroxysqualene	163.364
24	ZINC000008220036	2-Hexaprenyl-3-Methyl-6-Methoxy-1,4 Benzoquinone	162.62
25	ZINC000004096910	2-Hexaprenyl-6-Methoxyphenol	162.34
26	ZINC000004096888	Mesobilirubinogen	162.03
27	ZINC000049878510	N-Lignoceroyltryptamine	161.995
28	ZINC000004096892	Urobilinogen	161.85
29	ZINC000014951634	Endomorphin 2	161.81
30	ZINC000014952116	Enkephalin	160.642

### Prediction of the pharmacological properties of the candidate compounds

Safety should be involved and fully evaluated when screening potential drugs for the perspective possible toxicity. This study deeply examined different toxicity properties including rodent carcinogenicity, Ames mutagenicity, weight of evidence carcinogenicity, and developmental toxicity potential for the candidate compounds and the reference drug. According to the predictions of the TOPKAT module, results suggested that all compounds including the reference drug had developmental toxicity potential, 18 compounds showed no Ames mutagenicity (scores ≤0.1), 16 compounds had no (scores ≤0.1) and seven had low (scores 0.1–0.4) weight of evident carcinogenicity, and different compounds showed different rodent carcinogenicity potentials to rat or mouse. The detailed information on toxicity characteristics of each candidate compound was shown in **Table 2**.

**Table 2:** Toxicity calculation of the top 30 materials.

Number	Compounds	Rat NTP		Mouse NTP		Ames	WOE	DTP
		Male	Female	Male	Female			
1	ZINC000062238222	0.969	0.000	0.000	0.000	0.989	0.132	1.000
2	ZINC000085544839	0.964	0.000	0.080	0.000	0.999	0.057	1.000
3	ZINC000004096878	0.000	0.000	1.000	0.000	1.000	0.740	1.000
4	ZINC000085545908	0.000	0.000	0.000	1.000	0.000	0.000	1.000
5	ZINC000095620524	0.999	1.000	0.000	1.000	0.000	1.000	1.000
6	ZINC000004096653	0.000	1.000	1.000	0.000	1.000	0.000	1.000
7	ZINC000085541163	0.998	1.000	1.000	0.186	0.000	0.362	1.000
8	ZINC000004096684	0.000	1.000	1.000	0.000	1.000	0.000	1.000
9	ZINC000072131515	0.000	1.000	1.000	0.000	1.000	0.000	1.000
10	ZINC000004099069	0.000	0.000	0.000	0.000	0.002	0.000	0.864
11	ZINC000014712793	0.632	1.000	0.000	0.640	0.000	0.997	1.000
12	ZINC000150338786	0.000	0.000	1.000	0.000	1.000	0.719	1.000
13	ZINC000085826837	0.998	1.000	1.000	0.186	0.000	0.362	1.000
14	ZINC000004099068	0.000	0.000	0.000	0.000	0.002	0.000	0.864
15	ZINC000014951658	0.000	1.000	0.000	1.000	0.000	0.000	1.000
16	ZINC000044086691	0.998	0.987	0.000	1.000	0.004	0.499	1.000
17	ZINC000049878197	1.000	0.000	1.000	0.819	0.000	0.000	0.908
18	ZINC000085826835	0.998	1.000	1.000	0.186	0.000	0.362	1.000
19	ZINC000008552163	0.160	0.008	0.010	0.000	0.927	1.000	0.000
20	ZINC000008552069	0.997	0.000	0.001	0.031	0.024	0.142	1.000
21	ZINC000042805482	0.998	1.000	1.000	0.186	0.000	0.362	1.000
22	ZINC000013513540	0.840	0.020	0.000	0.139	0.000	0.248	1.000
23	ZINC000040976869	0.000	1.000	1.000	0.000	0.021	0.046	0.000
24	ZINC000008220036	0.000	1.000	1.000	0.000	0.064	0.000	0.000
25	ZINC000004096910	0.000	1.000	1.000	0.000	1.000	0.000	1.000
26	ZINC000004096888	0.000	0.000	0.000	0.855	1.000	0.000	1.000
27	ZINC000049878510	1.000	0.000	1.000	0.816	0.000	0.000	0.908
28	ZINC000004096892	0.000	0.000	0.000	1.000	1.000	0.000	1.000
29	ZINC000014951634	0.000	1.000	0.000	0.089	0.000	0.000	1.000
30	ZINC000014952116	0.000	0.000	0.000	0.001	0.000	0.000	0.928

NTP, NTP Carcinogenicity Call; Ames, Ames Mutagenicity; WOE, Weight of Evidence Carcinogenicity Call; DTP, Developmental Toxicity Potential.

The *in vivo* pharmacological properties of these compounds were also assessed based on the ADMET module, including aqueous solubility level, BBB penetration level, CYP2D6 binding level, hepatotoxicity level, human intestinal absorption level, and PPB level. As shown in **Table 3**, results represented four compounds including ZINC000072131515, ZINC000100045922, ZINC000008220036, and ZINC000004096910 that showed poor aqueous solubility, while others had moderate (scores 1–3) or high (scores >3) levels (defined in water at 25°C). All compounds had hypertonicity of BBB. Nine compounds were calculated to be toxic to the liver, and nine compounds had high intestinal absorption level. As for the reference drug, it was predicted to have no CYP2D6-binding property, to be toxic to the liver, and to have a high intestinal absorption level.

**Table 3:** Pharmacological properties of the top 30 materials based on ADME (Absorption, Distribution, Metabolism, and Excretion).

Number	Compound	Aqueous solubility ^a^	BBB Level ^b^	CYP2D6 ^c^	Hepatotoxicity ^d^	Intestinal absorption^e^	PPB prediction ^f^
1	ZINC000062238222	3	4	FALSE	TRUE	FALSE	FALSE
2	ZINC000085544839	3	4	FALSE	TRUE	FALSE	FALSE
3	ZINC000004096878	1	4	FALSE	TRUE	TRUE	TRUE
4	ZINC000085545908	3	4	FALSE	FALSE	FALSE	FALSE
5	ZINC000095620524	4	4	FALSE	TRUE	FALSE	FALSE
6	ZINC000004096653	1	4	FALSE	FALSE	TRUE	TRUE
7	ZINC000085541163	2	4	FALSE	FALSE	FALSE	FALSE
8	ZINC000004096684	1	4	FALSE	FALSE	TRUE	TRUE
9	ZINC000072131515	0	4	FALSE	FALSE	TRUE	TRUE
10	ZINC000004099069	3	4	FALSE	FALSE	FALSE	FALSE
11	ZINC000014712793	4	4	FALSE	FALSE	FALSE	FALSE
12	ZINC000150338786	1	4	FALSE	TRUE	TRUE	TRUE
13	ZINC000085826837	2	4	FALSE	FALSE	FALSE	FALSE
14	ZINC000004099068	3	4	FALSE	FALSE	FALSE	FALSE
15	ZINC000014951658	3	4	FALSE	FALSE	FALSE	FALSE
16	ZINC000044086691	1	4	FALSE	FALSE	TRUE	TRUE
17	ZINC000049878197	1	4	FALSE	FALSE	FALSE	FALSE
18	ZINC000085826835	2	4	FALSE	FALSE	FALSE	FALSE
19	ZINC000008552163	4	4	FALSE	FALSE	FALSE	FALSE
20	ZINC000008552069	4	4	FALSE	TRUE	FALSE	FALSE
21	ZINC000042805482	2	4	FALSE	FALSE	FALSE	FALSE
22	ZINC000013513540	4	4	FALSE	TRUE	FALSE	FALSE
23	ZINC000040976869	3	4	FALSE	FALSE	TRUE	TRUE
24	ZINC000008220036	0	4	TRUE	FALSE	TRUE	TRUE
25	ZINC000004096910	0	4	TRUE	FALSE	TRUE	TRUE
26	ZINC000004096888	2	4	FALSE	TRUE	FALSE	FALSE
27	ZINC000049878510	1	4	FALSE	FALSE	FALSE	FALSE
28	ZINC000004096892	2	4	FALSE	TRUE	FALSE	FALSE
29	ZINC000014951634	3	4	FALSE	FALSE	FALSE	FALSE
30	ZINC000014952116	4	4	FALSE	FALSE	FALSE	FALSE
31	Reference drug	/	/	FALSE	TRUE	TRUE	TRUE

ADME, Adsorption, Distribution, Metabolism and Excretion properties of compounds.

a Aqueous solubility level: 0 (extremely low); 1 (very low, but possible); 2 (low); 3 (good).

b Blood–brain barrier level: 0 (Very high penetrant); 1 (High); 2 (Medium); 3 (Low).

c Cytochrome P450 2D6 level: TRUE (Inhibitor); FALSE (Non-inhibitor); ^d^Hepatotoxicity: TRUE (Toxic); FALSE (Nontoxic); ^e^intestinal absorption level: TRUE (Absorbent strong); FALES (Absorbent weak); ^f^Plasma Protein Binding: TRUE (Absorbent strong); FALSE (Absorbent weak).

Considering the above pharmacological prediction results, five compounds (ZINC000040976869, ZINC000072131515, ZINC000062238222, ZINC000014951634, and ZINC000004099068) were further considered as the significant compounds with relatively low hepatoxicity, carcinogenicity, and Ames mutagenicity and high aqueous solubility and intestinal absorption levels, which were further employed for the following analysis.

### Analysis of the high-precision docking algorithm

**Figure 5:**
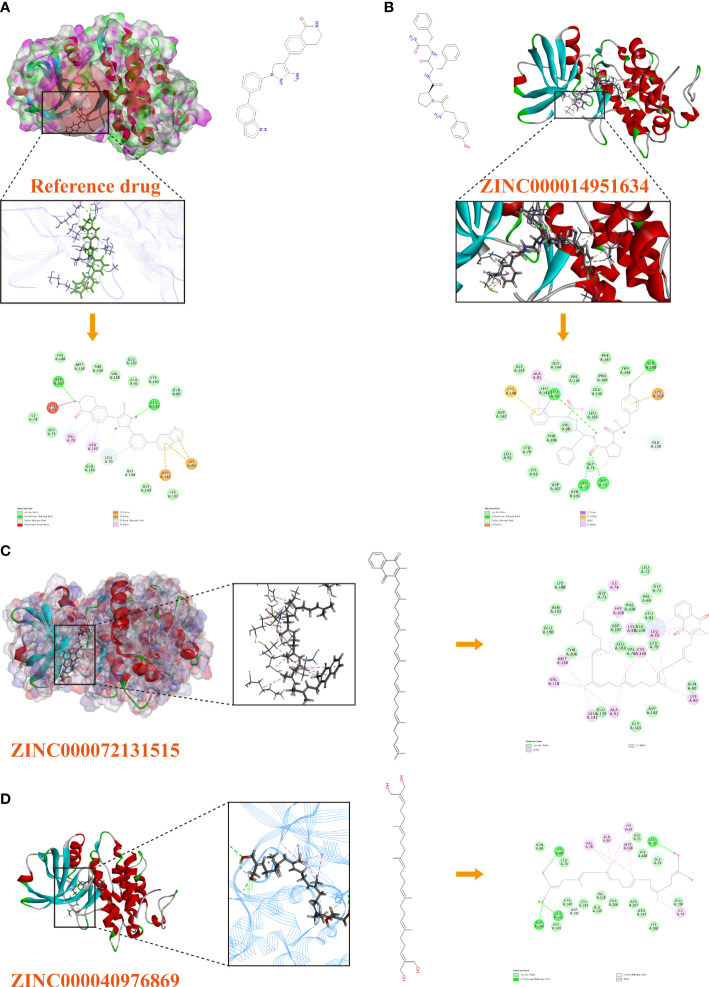
**(A)** The docking pattern illustrating the chemical bond interactions of the reference drug and MAPKAPK2. **(B)** The docking pattern illustrating the chemical bond interactions of ZINC000014951634 and MAPKAPK2. **(C)** The docking pattern illustrating the chemical bond interactions of ZINC000072131515 and MAPKAPK2. **(D)** The docking pattern illustrating the chemical bond interactions of ZINC000040976869 and MAPKAPK2.

Based on these five significant compounds, this study then studied the binding mechanisms between the ligands and *MAPKAPK2* receptor using the CDOCKER module, which was a more precise docking algorithm to generate more accurate chemical bond interactions. To prove the reliability of the CDOCKER module in this docking system, this study extracted the initial posture of the inhibitor from *MAPKAPK2* and then redocked into the binding pocket region. Results displayed that the RMSD between the initial crystal complex and the docked pose was 1.382 Å, suggesting the high reliability of the CDOCKER module. After precise docking algorithm, three compounds including ZINC000072131515, ZINC000014951634, and ZINC000040976869 docked successfully with the binding pocket region of *MAPKAPK2*. A total of 10 conformations of each compound were generated; the lowest energy of each ligand–*MAPKAPK2* complex was shown in **Table 4**. The CDOCKER interaction energy of ZINC000014951634–*MAPKAPK2* complex (-55.945 kcal/mol) was significantly lower than that of the reference drug–*MAPKAPK2* complex (-46.237 kcal/mol), and the other two complexes ZINC000072131515–*MAPKAPK2* (-45.449 kcal/mol) and ZINC000040976869–*MAPKAPK2* (-46.263 kcal/mol) had similar interaction energy values compared to the reference drug. The binding patterns of these three ligand–*MAPKAPK2* complexes and reference drug were shown in **Figures 5A–D**. Results illustrated that ZINC000014951634 generated four hydrogen bonds, three pi-alkyl bonds, two carbon bonds, one Pi-Cation bond, one Pi-Sigma bond, and an alkyl bond. ZINC000040976869 generated four hydrogen bonds, one carbon bond, and seven alkyl bonds with receptor *MAPKAPK2*. ZINC000072131515 formed one pi-alkyl bond and 16 alkyl bonds with receptor *MAPKAPK2*. As for the reference drug, it formed two hydrogen bonds, three pi-alkyl bonds, one carbon bond, two Pi-Cation bonds, and one Pi-donor bond with the receptor. Judging from these ligand structures, ZINC000072131515 and ZINC000040976869 had similar molecular structures, and ZINC000014951634 and the reference drug had similar structures. The detailed chemical bond information was displayed in **Table 5** and **Supplementary Table S3**, including the accurate atom interactions and chemical bond distances.

**Table 4:** CDOCKER interaction energy of the significant materials and the reference drug with MAPKAPK2.

Compounds	CDOCKER Interaction Energy (Kcal/mol)
ZINC000072131515	-45.449
ZINC000014951634	-55.945
weZINC000040976869	-46.263
Reference drug	-46.237

**Table 5:** Chemical bond interaction parameters of ZINC000014951634 and ZINC000040976869 ligands with MAPKAPK2 residues.

Receptor	Compound	Interaction residues	Distances (Å)	Chemical bond type
MAPKAPK2	ZINC000014951634	A:LEU72:HN - ZINC000014951634:O25	2.40	Hydrogen bond
		A:GLY73:HN - ZINC000014951634:O25	2.37	Hydrogen bond
		A:SER148:HN - ZINC000014951634:O40	2.70	Hydrogen bond
		ZINC000014951634:H62 - A:LEU70:O	2.95	Hydrogen bond
		A:GLY73:HA2 - ZINC000014951634:O25	2.87	Carbon bond
		ZINC000014951634:H70 - A:GLU190:OE1	3.05	Carbon bond
		A:LYS353:HZ3 - ZINC000014951634	2.75	Pi-Cation bond
		A:LEU70:HD22 - ZINC000014951634	2.25	Pi-Sigma bond
		ZINC000014951634 - A:LEU72	5.20	Alkyl bond
		ZINC000014951634 - A:ALA91	4.68	Pi- Alkyl bond
		ZINC000014951634 - A:CYS140	4.88	Pi- Alkyl bond
		ZINC000014951634 - A:LYS353	5.24	Pi- Alkyl bond
	ZINC000040976869	A:LYS89:HZ2 - ZINC000040976869:O34	2.69	Hydrogen bond
		A:GLY144:HN - ZINC000040976869:O32	2.35	Hydrogen bond
		ZINC000040976869:H73 - A:LEU72:O	2.74	Hydrogen bond
		ZINC000040976869:H81 - A:LEU141:O	2.29	Hydrogen bond
		ZINC000040976869:H81 - A:LEU141:O	2.85	Carbon bond
		A:ALA91 - ZINC000040976869:C7	4.40	Alkyl bond
		ZINC000040976869:C7 - A:VAL78	3.54	Alkyl bond
		ZINC000040976869:C7 - A:LYS93	4.04	Alkyl bond
		ZINC000040976869:C7 - A:MET138	4.38	Alkyl bond
		ZINC000040976869:C13 - A:VAL78	4.42	Alkyl bond
		ZINC000040976869:C13 - A:LYS93	4.94	Alkyl bond
		ZINC000040976869:C18 - A:ILE74	4.44	Alkyl bond

### Assessment of the molecular dynamics simulation

**Figure 6:**
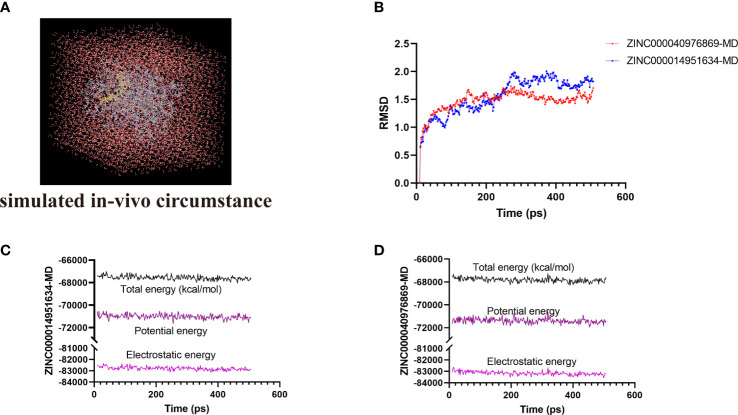
**(A)** Orthorhombic box with an explicit periodic boundary solvation water model. **(B)** Average backbone root mean square deviation (RMSD) of these two ligand–MAPKAPK2 complexes. **(C, D)** Different kinds of energy values of ZINC000014951634–MAPKAPK2 and ZINC000040976869–MAPKAPK2 complexes during molecular dynamics simulation.

The most optimal conformations of the two ligand–*MAPKAPK2* complexes (ZINC000014951634–*MAPKAPK2* and ZINC000040976869–*MAPKAPK2* complexes) were generated from the precise docking algorithm and were employed for the molecular dynamics simulation, which was conducted to simulate the *in vivo* circumstance to observe the stability of these two complexes during metabolic activity (**Figure 6A**). During the simulated activity *in vivo*, a total of 250 conformations were generated for each complex, with the initial conformation set as the reference, and the RMSD value was calculated based on the trajectory protocol. **Figure 6B** displayed that the RMSD values fluctuated smoothly. The different energy values of the two complexes generated from the *in vivo* activity process also demonstrated that each energy value, including total energy, electrostatic energy, and potential energy, was stabilized with time, as shown in **Figures 6C** and **D**, proving that the two compounds, ZINC000014951634 and ZINC000040976869, could bind with the receptor *MAPKAPK2* stably in the internal environment. Therefore, these two compounds were finally determined as the potential ideal inhibitors that could be targeted on *MAPKAPK2* to ameliorate OP.

## Discussion

OP has become one of the most common diseases according to the World Health Organization (WHO) report; nearly 50% of women suffer fracture pain due to the OP state (31). The early diagnosis of OP requires a high index of suspicion, since the bone loss is imperceptible, which is initially an asymptomatic process. Thus, OP is frequently diagnosed only after the first clinical fracture occurs (32, 33). Therefore, the common therapies mainly aim at preventing further fractures and relieving the pain. It is obvious that early detection and diagnosis are essential to evaluate the risk levels to make early intervention of OP.

Previous existing risk factors of OP included age, previous fragility fractures, low body mass index, frequent smoking, use of glucocorticoids, and history of fractures (34). While in recent decades, more reports have focused on the mechanisms, genetic alterations, and molecular and cellular activities: several key molecules coordinating the activities of osteoclasts and osteoblasts during bone remodeling have already created novel concepts in bone pathology and physiology (35). Moreover, several targeted drugs have also presented prospects in the treatment of OP, such as inhibitors targeting Receptor Activator of Nuclear Kappa-B Ligand (RANKL), protease cathepsin K, and src kinase (36–39). Although some of these inhibitors were effective, most still had limitations and side effects that may influence long-term administration and adherence (40). Consequently, discovering more specific biomarkers of OP and finding novel natural inhibitors targeting these macromolecules are still an urgent need for researchers to resolve.

This study first discovered potential interested modules responsible for the progression of OP through the WGCNA method, which is a powerful analytic algorithm based on holistic data mining (41). The application of WGCNA has been widely used in a variety of diseases, such as blood disease, disc degeneration, and neoplasms (42–44), which builds a bridge between sample characteristics and gene expression matrix. A total of 16 distinct co-expression modules were generated according to WGCNA in this study, among which the brown module was determined as the key module in the development of OP, with the highest correlations and lowest P value. The module–trait correlation and eigengene adjacency heatmap validated our results, and hierarchical clustering analysis also illustrated that the active genes in the brown module could distinguish the OP group from the normal group significantly. Further GO, KEGG, and GSEA results provided more understanding of the specific functions of these active genes in the progression of OP. Several OS-related functions were analyzed, such as cellular response to OS, misfolded protein binding, and response to ER stress; several abnormal signaling pathways of OP such as relaxin signaling pathway and *MAPK* and *TNF* signaling pathway were also found related to OS in the development of OP, which provided evidence for the essential roles of OS-related genes (45). Consequently, this study further determined the hub OS-related genes of OP in the following research to provide more potential biomarkers whether for diagnosis or targeted therapy.

After differential analysis of OP, 440 DEGs were identified, with 212 upregulated genes and 228 downregulated genes. The hub OS-related genes (DEOSGs) were finally determined by the intersection of genes in the brown module, the DEGs, and the obtained OS-related genes to generate more accurate results. Ultimately, 12 genes were selected as the DEOSGs of OP, including *PPP1R15A*, *CYB5R3*, *BCL2L1*, *ABCD1*, *MAPKAPK2*, *HSP90AB1*, *CSF1*, rela, *P4HB*, *AKT1*, *HSP90B1*, and *CTNNB1*. More research could be focused on these potential active genes whether for diagnosis or targeted therapy.

By consulting the literature, some studies reported their inhibitor research regarding *MAPKAPK2*, indicating that the biological functions of inhibiting *MAPKAPK2* have been confirmed. While the research on natural inhibitors of *MAPKAPK2* (namely, *MK2*) remained few, which blocked the discovery and reservation of compounds regarding *MAPKAPK2* in the pharmacological market. *MAPKAPK2* was reported to be the target by inhibiting the *p38MAPK*–*MK2* axis to ameliorate chemotherapy-induced senescence-associated secretory phenotype and thereby maintain bone integrity in chemotherapy-treated mice (46). Thus, this study further focused on *MAPKAPK2* to discover more potential natural materials. It is also worth noting that in addition to *MAPKAPK2*, other hub genes listed in this study can also be considered as biological macromolecules and conducted for virtual screening. More targeted virtual docking analyses could be carried out in the future against a series of hub genes provided in this study.

Altogether, 18,225 natural materials were put into the ligand-binding pocket for high-throughput screening. The higher docking scores indicated more stable docking conformation of the complex. A total of 10,075 compounds could bind to the active site, among which 276 compounds had higher docking scores than the reference drug. Based on the ordering of docking scores, the top 30 compounds were selected as candidate compounds and their pharmacological properties were assessed using ADMET predictions. After the evaluation of these characteristics, this study further screened five compounds as the significant natural materials, with relatively low hepatoxicity, carcinogenicity, and Ames mutagenicity and high aqueous solubility and intestinal absorption levels, which had safer properties than the reference drug (predicted to be toxic to the liver). It is worth noting that although some of the candidate drugs on the list had certain side effects such as carcinogenicity, Ames mutagenicity, and developmental toxicity potential, the properties could also be improved by editing certain atoms or pharmacophores. In brief, this study provided a group of candidate compounds with different characteristics. Future drug tests or modifications could be focused on these drugs to make more experiments.

Subsequently, we analyzed the certain chemical bond interactions between the significant compounds and *MAPKAPK2* protein. Results showed that only three compounds could dock at the binding pocket region of *MAPKAPK2* based on the precise docking algorithm CDOCKER module, namely, ZINC000072131515 (vitamin K2), ZINC000014951634 (endomorphin-2), and ZINC000040976869 (tetrahydroxysqualene). Results displayed that these compounds had similar or even lower interaction energy than the reference drug, elucidating the high binding affinity with the receptor in real situations. Through chemical bond analysis of these complexes, these three ligands formed more bonds with the receptor, indicating stronger interactions, which could have more opportunities to dock at the regulatory sites and thereby inhibit the *MAPKAPK2* functions. Among them, ZINC000072131515 was a widely reported drug that could inhibit the progression of OP, and this study further elucidated that ZINC000072131515 was at a certain extent a natural inhibitor material to ameliorate OP progression, at least by targeting *MAPKAPK2* and thereby inhibiting its functions. ZINC000014951634 and ZINC000040976869 both displayed different biological activity roles in other diseases. Basically, the similar structures of the compounds resulted in similar logical functions. Judging from these ligand structures, ZINC000072131515 and ZINC000040976869 had similar molecular structures, and ZINC000014951634 and the reference drug had similar structures. We could hypothesize the similar functions of ZINC000040976869 and ZINC000014951634, like ZINC000072131515 and the reference drug did.

Ultimately, we put the two complexes, ZINC000014951634–*MAPKAPK2* and ZINC000040976869–*MAPKAPK2*, into a simulated *in vivo* circumstance to evaluate the stability of these two complexes through molecular dynamics simulation. The RMSD and energy curves were plotted by calculating different values of these ligand–*MAPKAPK2* complexes. Results suggested that these chemical bonds could contribute to the stability of these complexes, including π-related interactions, hydrogen bond interactions, and alkyl interactions. The two complexes could exist stably under simulated *in vivo* circumstances and thereby regulate the activity of *MAPKAPK2*, which further proved the reliability of the ideal lead materials selected in this study.

The discovery of novel lead materials is always a key step for future drug design and development, which offers significant skeletons with biological activities. This study provided a group of candidate compounds for researchers. Future prospective drug improvement, such as pharmacophore modification and refinement, could be conducted, focusing on the novel natural skeletons. In conclusion, this study found a group set of OS-related biomarkers, providing further insights for OS functions in the development of OP. Then, focusing on one of the macromolecules, *MAPKAPK2*, to further discover potential novel materials, which was of great significance in guiding the screening of *MAPKAPK2* potential materials. In addition to *MAPKAPK2*, more targeted virtual docking analyses could be carried out in the future against a series of hub genes provided in this study.

## Conclusion

This study aimed to find novel OS-related biomarkers of OP and then focused on one of the macromolecules, *MAPKAPK2* protein, to further discover potential novel materials based on advanced structural biology approach. A total of 12 genes were selected as the DEOSGs of OP in this study, including *PPP1R15A*, *CYB5R3*, *BCL2L1*, *ABCD1*, *MAPKAPK2*, *HSP90AB1*, *CSF1*, *RELA*, *P4HB*, *AKT1*, *HSP90B1*, and *CTNNB1*. Then, based on the *MAPKAPK2* protein, two novel natural materials, namely, ZINC000014951634 and ZINC000040976869, were screened as ideal lead compounds targeting *MAPKAPK2*, with moderate safety and stable docking conformations. In brief, this study provided the pharmacological property information of the candidate compounds, together with valuable natural materials for further *MAPKAPK2*-targeted inhibitor research.

## Data availability statement

Publicly available datasets were analyzed in this study. This data can be found here: https://www.ncbi.nlm.nih.gov/geo/, GSE35958.

## Author contributions

This study was completed with teamwork. Each author had made corresponding contribution to the study. Conceived the idea: BG and WL; Manuscript draft: YZh, WL, and KZ; Software use: YZh, WL, YZo, XQ, and TL; Downloaded and collected data: YZo, XQ, and TL; Analyzed the data: YZh, WL, KZ, and MX; Prepared figures: BG, WL, YZh, YZo, and MX; Redressed the manuscript: BG, WL, and YZh; Reviewed the manuscript: All authors. All authors have read and approved the final manuscript.

## Funding

This study was supported by grants from the National Natural Science Foundation of China (NSFC, 82172475); and “the National Science Fund for Excellent Young Scholars” (NSFC, 82222046).

## Conflict of interest

The authors declare that the research was conducted in the absence of any commercial or financial relationships that could be construed as a potential conflict of interest.

## Publisher’s note

All claims expressed in this article are solely those of the authors and do not necessarily represent those of their affiliated organizations, or those of the publisher, the editors and the reviewers. Any product that may be evaluated in this article, or claim that may be made by its manufacturer, is not guaranteed or endorsed by the publisher.

## Supplementary material

Supplementary Table 1 The OS-related genes obtained from GeneCards database.

Supplementary Table 2 All the docked materials ranked by docking scores.
