# Supplementary material for: Revealing oxidative stress-related genes in osteoporosis and advanced structural biological study for novel natural material discovery regarding MAPKAPK2
Source: Front Endocrinol (Lausanne). 2022 Nov 21;13:1052721. doi: 10.3389/fendo.2022.1052721 (PMC9720258; doi:10.3389/fendo.2022.1052721)
Supplement: Supplementary file 3 [file DataSheet_3.docx]

**Supplementary Table 3.** Chemical bond interaction parameters of each compound with MAPKAPK2 residues.

| Receptor | Compound | Interaction residues | Distances (Å) | Chemical bond type |
| --- | --- | --- | --- | --- |
| MAPKAPK2 | Reference drug | A:LYS89:NZ - MAPKAPK2 | 4.41 | Pi-Cation bond |
|  |  | A:LYS89:NZ - MAPKAPK2 | 3.52 | Pi-Cation bond |
|  |  | A:GLY144:HN - MAPKAPK2 | 3.26 | Pi-Donor bond |
|  |  | MAPKAPK2 - A:LEU70 | 5.31 | Pi-Alkyl bond |
|  |  | MAPKAPK2:H33 - A:LEU141:O | 2.31 | Hydrogen bond |
|  |  | MAPKAPK2:H35 - A:LEU70:O | 2.82 | Carbon bond |
|  |  | MAPKAPK2 - A:LEU193 | 4.91 | Pi-Alkyl bond |
|  |  | MAPKAPK2 - A:VAL78 | 3.99 | Pi-Alkyl bond |
|  |  | MAPKAPK2:H39 - A:ASP207:OD2 | 2.28 | Hydrogen bond |
|  | ZINC000072131515 | ZINC000072131515:C3 - A:ILE74 | 4.49 | Alkyl bond |
|  |  | ZINC000072131515:C8 - A:LYS93 | 4.68 | Alkyl bond |
|  |  | A:HIS108 - ZINC000072131515:C8 | 4.95 | Pi-Alkyl bond |
|  |  | ZINC000072131515:C8 - A:MET138 | 5.05 | Alkyl bond |
|  |  | ZINC000072131515:C13 - A:VAL118 | 4.44 | Alkyl bond |
|  |  | ZINC000072131515:C13 - A:MET138 | 4.35 | Alkyl bond |
|  |  | A:ALA91 - ZINC000072131515:C13 | 3.05 | Alkyl bond |
|  |  | ZINC000072131515:C13 - A:LEU141 | 4.49 | Alkyl bond |
|  |  | ZINC000072131515:C18 - A:LEU70 | 4.75 | Alkyl bond |
|  |  | ZINC000072131515:C18 - A:CYS140 | 4.00 | Alkyl bond |
|  |  | A:ALA91 - ZINC000072131515:C18 | 3.23 | Alkyl bond |
|  |  | ZINC000072131515:C23 - A:LEU70 | 4.46 | Alkyl bond |
|  |  | ZINC000072131515:C23 - A:LYS89 | 4.69 | Alkyl bond |
|  |  | ZINC000072131515:C23 - A:CYS140 | 3.65 | Alkyl bond |
|  |  | ZINC000072131515:C33 - A:LEU70 | 4.29 | Alkyl bond |
|  |  | ZINC000072131515 - A:LEU70 | 4.15 | Alkyl bond |
|  |  | ZINC000072131515 - A:LEU70 | 5.48 | Alkyl bond |
